# Healthcare professionals' views about how pregnant women can benefit from using a closed‐loop system: Qualitative study

**DOI:** 10.1111/dme.15072

**Published:** 2023-03-07

**Authors:** Julia Lawton, David Rankin, Sara Hartnell, Tara Lee, Anna R. Dover, Rebecca M. Reynolds, Roman Hovorka, Helen R. Murphy, Ruth I. Hart, the AiDAPT Collaborative Group

**Affiliations:** ^1^ Usher Institute, Medical School, University of Edinburgh Edinburgh UK; ^2^ Cambridge University Hospitals NHS Foundation Trust Cambridge UK; ^3^ Norwich Medical School Norwich UK; ^4^ Norfolk & Norwich University Hospital NHS Foundation Trust Norwich UK; ^5^ Edinburgh Centre for Endocrinology and Diabetes, Royal Infirmary of Edinburgh Edinburgh UK; ^6^ Centre for Cardiovascular Science University of Edinburgh, Queen's Medical Research Institute Edinburgh UK; ^7^ Wellcome Trust‐MRC Institute of Metabolic Science, University of Cambridge Cambridge UK; ^8^ Department of Paediatrics University of Cambridge Cambridge UK

**Keywords:** closed‐loop system, continuous glucose monitoring, qualitative research, healthcare professionals, pregnancy, technology, type 1 diabetes

## Abstract

**Background:**

Interest is growing in how closed‐loop systems can support attainment of within‐target glucose levels amongst pregnant women with type 1 diabetes. We explored healthcare professionals' views about how, and why, pregnant women benefitted from using the CamAPS FX system during the AiDAPT trial.

**Methods:**

We interviewed 19 healthcare professionals who supported women using closed‐loop during the trial. Our analysis focused on identifying descriptive and analytical themes relevant to clinical practice.

**Results:**

Healthcare professionals highlighted clinical and quality‐of‐life benefits to using closed‐loop in pregnancy; albeit, they attributed some of these to the continuous glucose monitoring component. They emphasised that the closed‐loop was not a panacea and that, to gain maximum benefit, an effective collaboration between themselves, the woman and the closed‐loop was needed. Optimal performance of the technology, as they further noted, also required women to interact with the system sufficiently, but not excessively; a requirement that they felt some women had found challenging. Even where healthcare professionals felt that this balance was not achieved, they suggested that women had still benefitted from using the system. Healthcare professionals reported difficulties predicting how specific women would engage with the technology. In light of their trial experiences, healthcare professionals favoured an inclusive approach to closed‐loop rollout in routine clinical care.

**Conclusions:**

Healthcare professionals recommended that closed‐loop systems be offered to all pregnant women with type 1 diabetes in the future. Presenting closed‐loop systems to pregnant women and healthcare teams as one pillar of a three‐party collaboration may help promote optimal use.

**Novelty Statement**

- This is the first study to consult healthcare professionals with direct (≥6 months) experience of supporting closed‐loop use in pregnancy.
- Healthcare professionals perceived closed‐loop as conferring clinical and quality‐of‐life benefits.
- They observed that, to gain maximum benefit, an effective, three‐way collaboration between themselves, the woman and the closed‐loop was needed.
- They suggested that, ideally, women should interact with the system sufficiently but not excessively; however, even when this did not happen, they still felt women benefitted from using closed‐loop.
- As they experienced difficulties determining how specific women would engage with the technology, they favoured an inclusive approach to closed‐loop rollout.

## 1 INTRODUCTION

For optimal obstetric and neonatal outcomes, pregnant women with type 1 diabetes are advised to attain glucose levels of 3.5–7.8 mmol/L for at least 70% of the time.^1^ However, due to nausea, vomiting and physiological changes resulting in increased insulin sensitivity in early pregnancy and increased insulin resistance as pregnancy progresses, achieving and maintaining these pregnancy glucose targets can be challenging and psychologically demanding.^2^, ^3^

Interest is growing in how technology can support glycaemic management in pregnancy. Use of Continuous Glucose Monitoring (CGM) has been associated with improved antenatal glucose levels and neonatal health outcomes,^4^, ^5^ and CGM is now recommended for all pregnant women with type 1 diabetes in the UK.^6^, ^7^, ^8^ Hybrid closed‐loop systems (HCL), which link CGM with insulin pumps (automating delivery of basal insulin), have also been shown to offer glycaemic and/or psychosocial benefits to other user groups.^9^, ^10^, ^11^, ^12^ Until recently, however, research on HCL use in pregnancy has been limited to early‐phase studies of prototype systems involving small numbers of women.^13^, ^14^, ^15^, ^16^ Findings have been promising, demonstrating the safety and feasibility of HCL use by pregnant women. Associated qualitative research has suggested that the psychosocial impacts of HCL technology use may be somewhat mixed, with women reporting both benefits and burdens.^17^ To date, no studies have explored the perspectives of healthcare professionals who have supported pregnant women using HCL technology. This is an important omission, as these individuals can play key roles in determining who gets access to diabetes technologies^18^, ^19^ and how these technologies are subsequently experienced and used.^20^, ^21^

The AiDAPT trial is an open‐label, multi‐centre, randomised two‐arm trial comparing HCL with standard insulin delivery in pregnant women with type 1 diabetes.^22^ As part of a broader evaluation which included exploration of women's perspectives and experiences (forthcoming), we conducted interviews with healthcare professionals who provided care and support to pregnant women using the HCL during the trial. Key aims of the interview study were to explore healthcare professionals' views about: how and why pregnant women can benefit from HCL use; who benefits (most) from using a HCL and why; and who should be prioritised for use of the technology. Our objectives were to provide recommendations to inform decisions about rollout and use of HCL technology amongst pregnant women in routine clinical care.

## 2 METHODS

### 2.1 Overview

We conducted in‐depth interviews, enabling healthcare professionals to raise issues they considered salient, including those unforeseen at the trial outset. Our orientation was inductive, and we took an iterative approach to data collection and analysis. Our approach to reporting (of both methods and findings) has been informed by the consolidated criteria for reporting qualitative studies (COREQ).^23^ Research Ethics (18/EE/0084) and governance approvals were obtained in conjunction with approvals for the wider trial.

### 2.2 Setting/context

The AiDAPT trial (ISRCTN: 56898625) was conducted in the UK between 2019 and 2022.^22^ Eligibility criteria included having an HbA1c level of ≥48 mmol/mol (≥6.5%) at booking (first antenatal contact) and ≤86 mmol/mol (≤10%) at randomization, plus a willingness to use the study devices. More information on the HCL used (the CamAPS FX system) and its distinctive features is provided in Box 1. Women used the HCL for approximately 24 weeks of their pregnancy (weeks 13–37). The trial recruited 124 women, of whom 61 were randomized to HCL; staff reports indicate three women chose to discontinue HCL in the first few weeks of use.

BOX 1 **The CamAPS FX system.**

The CamAPS FX system links real‐time CGM technology, the Dexcom G6 (Dexcom, San Diego, CA, USA), with an insulin pump, the DANA RS (Sooil, Seoul, South Korea) via a **control algorithm, the CamAPS FX app** (CamDiab, Cambridge, UK), hosted on an unlocked Android smartphone (Galaxy S, Samsung, South Korea) running Android 8 OS or above.

The app/smartphone communicates wirelessly with both the CGM sensor and insulin pump, subject to being kept within 5–10 metres range of those devices. It uses CGM sensor data to direct (basal/background) insulin delivery via the pump, adjusting this automatically every 8–12 minutes. Users need to administer pre‐meal insulin boluses, via the app.

The app is also used to: (1) set personal glucose targets, typically 5.5 mmol/L in early pregnancy and 5.0 mmol/L after 14–16 weeks, consistent with achieving and maintaining pregnancy glucose targets; (2) temporarily increase or decrease (using ‘Boost’ or ‘Ease‐off’ settings) the rate of automated insulin delivery by ~33%, determined using real‐time CGM data. Participants received advice during closed‐loop training about when they might use these functions, and to use them at their discretion and/or following input from healthcare professionals; (3) personalise alarms alerting users to high/low glucose levels; and, (4) view data including CGM glucose levels, rate of insulin delivery and summary statistics. The app automatically uploads this data to the Diasend platform (Glooko/Diasend, Göteborg, Sweden), enabling data‐sharing with healthcare professionals and supporting remote monitoring throughout pregnancy.

Healthcare professionals delivering the trial had experience of working with pregnant women with type 1 diabetes and received training in use of the HCL and constituent devices. As well as delivering the trial, these individuals provided women's routine clinical care. Trial participants received the same type and frequency of clinical contacts as was given to pregnant women in routine clinical care. This typically comprised weekly/fortnightly (face‐to‐face or virtual) appointments to assess and optimise glucose levels and provide education, information and other (e.g., psychological) support.

### 2.3 Participants and recruitment

We recruited healthcare professionals from eight sites, after they had ≥6 months' experience supporting women using the HCL. We targeted individuals at each site who were heavily involved in providing HCL care and support to women, and sampled purposively to ensure diversity with respect to different grades and types of staff across the sites. Recruitment continued until we reached data saturation (where additional data did not add to our findings).

### 2.4 Data collection

Telephone interviews lasting 1–2 h were conducted by DR—a non‐clinical researcher with extensive experience of qualitative research—between June 2021 and April 2022. Interviews were informed by a topic guide (see Box 2) developed in light of earlier research exploring healthcare professionals' views about HCL use^19^, ^24^ and with input from clinical colleagues. We revised the guide, between interviews, in response to emergent findings. All interviews were digitally recorded and transcribed verbatim.

BOX 2 **Main topics explored in interviews (relevant to the analysis).**

- Interviewee's clinical background, current role and experience supporting people (including pregnant women) with type 1 diabetes.
- Experiences of: supporting pregnant women using CGM or flash monitoring technology; remote monitoring in routine (pregnancy) care; supporting women using HCL systems other than the CamAPS FX.
- Views on the challenges and burdens of managing type 1 diabetes in pregnancy.
- Experiences of recruiting into the AiDAPT trial; views about women who declined to take part or withdrew from the trial.
- Views about: the impact of the HCL system on women's diabetes self‐management practices; the benefits and drawbacks of women using HCL technology compared to other regimens (e.g., pump and multiple daily injections).
- Perspectives on women's: engagement with data; use of ‘Ease‐off’ and ‘Boost’ features; administration of correction doses.
- Experiences of: providing pregnancy‐related support and helping adjust settings/ratios for women using a HCL system; having remote access to women's real‐time data (e.g., perceived advantages/disadvantages).
- Experiences of contact initiated by women seeking support/input to optimise glucose levels.
- Views about: which types of women did (and did not) gain clinical benefit from using the HCL; reasons why women did more or less well than expected when using the HCL.
- Views about who should be given access to HCL technology in routine care.

### 2.5 Data analysis

Data analysis focused on identifying descriptive and analytical themes^25^ with relevance to clinical practice. Hence, the work was guided by both a priori and emergent interests. Three experienced qualitative researchers (JL, DR and RIH) were involved in the analysis, which entailed reading transcripts repeatedly and using the method of ‘constant comparison’^26^ to identify initial, cross‐cutting themes. A coding frame was then developed to capture these themes and associated data. We used the qualitative software package Nvivo 11 (QSR International) to facilitate data coding and retrieval. Coded datasets were subjected to further in‐depth analyses to inform identification of sub‐themes and illustrative quotations. Throughout the analytical process, team members reviewed data independently and wrote separate analytical reports before meeting to discuss their interpretations and reach agreement on key themes.

## 3 RESULTS

We interviewed 19/22 healthcare professionals responsible for providing care and support to women using HCL during the trial (see Table 1 for further information about the sample). In aggregate, these individuals had supported approximately two‐thirds of women using HCL during the trial.

**TABLE 1:** Characteristics of the sample.

	*N* (%)^a^
AiDAPT sites (*n* = 8)	
Total number of interviewees	19
Interviewees per site: range (mode)	1–4 (3)
Role	
Diabetes Consultants/doctors	11 (57.9)
Nurse Consultants	2 (10.5)
Diabetes Specialist Nurses	4 (21.1)
Dietitian	1 (5.3)
Diabetes Specialist Midwife	1 (5.3)
Years of diabetes experience	
5–10 years	4 (21.1)
10–20 years	5 (26.3)
>20 years	10 (2.6)
Interviewees with previous experience supporting HCL users (during trials or in routine care)	12 (63.2)
Gender	
Female	16 (84.2)
Male	3 (15.8)
Age in years: mean, SD (range)	48.7 ± 7.1 (33–60)

^a^ Percentages may not sum to 100% due to rounding.

Key themes arising from the analysis included: distinctive benefits to using CGM; added benefits to using the HCL (less work, but still work); collaboration (being) a condition for maximum HCL benefit; and, candidacy and (difficulties) predicting who gains (most) benefit from HCL use. These themes (and associated sub‐themes) are reported below, before we consider healthcare professionals' views about who should be encouraged to use an HCL in routine clinical care. As interviewees' perspectives on these topics were broadly consistent, we have not separated out our reporting according to their clinical role/background.

### 3.1 Distinctive benefits to using CGM

Interviewees described wide‐ranging clinical and quality‐of‐life benefits arising from HCL use in pregnancy. In doing so, however, they highlighted benefits attributable to using CGM rather than HCL technology per se. Several described CGM as being the “game‐changer” (HP‐001, HP‐003, HP‐011) in (type 1) diabetes pregnancy management. As well as providing women and themselves with better information to inform diabetes management decisions (e.g., dietary choices, titration of insulin doses) (see Table 2), interviewees reported how, by alerting women to out‐of‐range glucose levels, CGM helped them feel more confident working towards tight(er) pregnancy glucose targets:

**TABLE 2:** Additional participant quotations.

Themes and sub‐themes		Participant quotations
CGM alone offers important benefits (… but may be a source of anxiety)		“what CGM has, does, is it … means that women can understand the impacts of their food choices on their day‐to‐day glycaemia … And then the CGM gives us much information around the impact of the food and lifestyle choices on glycaemia, that helps us to customise the insulin doses that you can to the particular person in front of you.” (HP‐014) “it enables, well, it should enable, the visualisation of results in a way that allows them to make changes themselves … the simple things like being able to see what's happening overnight. So you are able to change your insulin levels overnight … whereas before, we, we could not do that. Or it was much more difficult to do that. You were basing your overnight values on what was happening in the morning, and that was often a crude – so it improves the, the granularity of the things, the decisions that we are making.” (HP‐006) “well, ‘cause the targets they are aiming for are so tight, it's really hard – or impossible – to achieve what we are aiming at. I think being pregnant's really difficult anyway, ‘cause you feel this sort of sense of responsibility towards your unborn child … I think sometimes the CGM is a kind a of double‐edged sword, cause suddenly you are aware of all your blood sugars, and every time your blood sugar's 12, you think, ‘Am I gonna harm my baby?’” (HP‐016)
Added value of the HCL: less work … but still work		“I think they are totally transformational for women, not just in terms of glucose control, but in terms of quality of life.” (HP‐010) “the majority of women do really, really well … they are over 70% time‐in‐range, and they love it, because to achieve that, they have not had to work as hard.” (HP‐018) “what they are having to do is so much less, as well … it's less work for them definitely, while achieving better control.” (HP‐017) “one of the women said to us, in clinic … ‘(I) wake up in the morning and the first thing I think about is not diabetes, and this has been the first time in my life that I've been able to do that’.” (HP‐003) “it's not a fix (for) everything … this does not mean that you are not longer driving your diabetes. It's a case of being able to … take your eye off the ball, just a little bit. But … it does not take care of everything … you have not actually got an artificial pancreas … it's only as good as the person who's using (it).” (HP‐005)
Collaboration: a condition of maximum benefit		“… making sure that if they are using the ‘Ease‐off’, that they are using it for a good amount of time, and checking what their basal is doing before they switch it on – ‘cause if it's hardly giving any insulin anyway, (there)'s not really much point!” (HP‐002) “The issue … is the knowing when to use the ‘Boost’ and the ‘Ease‐off’ … sometimes that's an issue. And sometimes women are just boosting when perhaps they … should be looking at their bolus doses, rather than just giving a dose and then giving a boost.” (HP‐009) “where it does not work quite so well is where I suppose there's lack of engagement. So if you are not bolusing at the correct time, or you ignore your alarms … it cannot do everything … And then there's the patient who does not trust the system and is micro‐managing … they'll start boosting and everything when perhaps it needs you to let the pump do its job. But they … just cannot let go … So there's sort of two types where it's not working well.” (HP‐015) “When people aren't too controlling … the better they do, because the pump can do its thing, the algorithm can do its thing. But yeah, that's quite hard … when they are pregnant.” (HP‐001)
Respecting the HCL's role and trusting it to do its job		“We did have one woman who was probably using ‘Boost’ too often… which just did not really allow the algorithm to learn very well, because she was boosting all the time. So we discouraged it, and she seemed to settle down.” (HP‐007)
Candidacy and the provisioning of HCL technology in routine care	Difficulties predicting who would benefit	“I'm surprised how quickly a couple of people that went on it – they just took off with it. Like one woman who's a bit flaky, but ever so nice, she went on it. And we … every time we speak, she says, ‘Oh fine, brilliant’. She just took to it straightaway, and that, that was it.” (HP‐016) “the biggest limitation to using it (the HCL) … will be mis‐timed, or not given, or mis‐calculated boluses. The closed‐loop will tend to mop that up a little bit, but never as much as people think.” (HP‐014)
	Use of HCL in routine care	“I'm really hopeful they'll show benefit and … we can use it with loads more pregnant women with type 1. We can offer it … that's what I would like, you know, like we can with Dexcom. We can offer it to everyone with type 1. I'd love to be able to offer this as well.” (HP‐001) “People who have got to the point of managing their own diabetes by tinkering with everything themselves, I do think that the closed‐loop, because it's part automated, does not do as well for them … They essentially have been running themselves like an insulin pump, without having a pump.” (HP‐007) “One would hope it would be any type 1 patient would be entitled to use a closed‐loop system for the duration of their pregnancy. I would hope they would not put a “too good control” limiting factor on it, but they might … One would hope it would be equal access for everyone.” (HP‐015)

“I think the CGM's made such a difference to people in preventing hypos… and that means that we can be a bit more aggressive with the insulin changes, because you're less worried about hypos, particularly in the first trimester that are a real concern.” (HP‐002)

Interviewees also noted, however, how some women had found having access to a constant stream of data overwhelming, with awareness of out‐of‐range readings heightening their anxiety and sometimes causing unwarranted distress (see Table 2).

### 3.2 Added benefits to using the HCL: less work but still work

Interviewees observed how the HCL provided additional benefits, by taking on (some of) the work of diabetes management (see Table 2). Specifically, they praised the HCL for handling (automating) administration of basal insulin, delivering this predictively, based on algorithmic learning, and in response to CGM information, with the goal of keeping glucose levels as close as possible to personal/user‐specified targets:

“I think it's really great in pregnancy, because it fulfils that really unique role, where things change on a day‐to‐day, or week‐to‐week basis … having a little bit taken over and automated, so the women don't have to think about it, and worry about it, makes a huge difference.” (HP‐018)

“it seems to just take away the complexities of the hour‐to‐hour glucose management that we, that often women struggle with … the closed‐loop takes some of that pressure away.” (HP‐007)

In addition, some described how knowing that the HCL would suspend basal insulin delivery if glucose fell below a specified threshold alleviated women's worries about hypoglycaemia and reduced the psychological burden of diabetes:

“it does improve … quality of life in pregnancy, it's one less thing for them to have to worry about. Well, they still worry about it, but not as intently.” (HP‐019)

Importantly, however, interviewees stressed that the HCL was not a panacea, and to gain maximum glycaemic benefit, a lot of work was still required:

“you still have to do stuff to really get the … extraordinary control that is possible … it's definitely not just plug it in and leave it… it's the Aston Martin of the pump world, but you still need someone to drive it.” (HP‐004)

### 3.3 Collaboration: a condition for maximum HCL benefit

Interviewees noted how achieving maximum benefit was contingent upon establishment of an effective three‐way collaboration involving the healthcare team, women and the HCL. Reflecting on their own role in this partnership, interviewees stressed the healthcare team's responsibility to provide women with comprehensive support:

“if we're going to look at offering these systems more widely, (we) would have to offer the package … it isn't just, you know, plug in and play. You don't just say, ‘Here you go, here's a pump and the app, and bye‐bye’. You have to give them that support.” (HP‐010)

They described how this support package typically included: pregnancy‐specific diabetes‐management advice; information about different features of the HCL, e.g., when and how to use the ‘Boost’ and ‘Ease‐Off’ functions (see Table 2), how to access and interpret data displayed in the app and on Diasend (Glooko/Diasend, Göteborg, Sweden); and, regular review and updating of certain settings, in particular meal‐time insulin‐to‐carbohydrate ratios (ICR).

Turning to women's role, interviewees emphasised the importance of them, firstly, engaging with their clinical team (e.g., by attending appointments and following instructions and advice to alter meal‐time ICR), and, secondly, with the healthcare team's support, creating conditions under which the HCL could perform optimally. The latter, they suggested, required women to attend carefully to dietary choices, count carbohydrates accurately, and administer accurate pre‐meal boluses at the correct times:

“I try to stress to them that … to get the most out of using closed‐loop, it's almost like when … they first started taking insulin and they were very careful about the timings of it, and careful about their eating, that the closed‐loop will work best under those sort of scenarios.” (HP‐014)

Interviewees further observed how effective collaboration with the HCL involved women interacting with the system appropriately – that is, sufficiently, but not excessively (see Table 2). This, as they suggested, required women to know and understand when it was helpful to intervene (for instance, by using the ‘Boost’ function) and when to allow the HCL to operate automatically. Indeed, some suggested that effective HCL use required women to tread a delicate line between retaining and delegating glycaemic management tasks to the HCL:

“I think probably the optimal psychological approach is to trust the system enough. So it's being a bit relaxed about the diabetes, but not too relaxed. And that's a really difficult balance. So not interfering with it too much, letting it get on and do its thing. But still being very engaged with your diabetes to make sure you're giving the boluses and all those kind of things.” (HP‐003)

### 3.4 Respecting the role of the HCL and trusting it to do its job

While interviewees suggested that entrusting glycaemic management to the system could present difficulties for any user, they noted that the high‐stakes nature of diabetes management in pregnancy could amplify this challenge (see Table 2). In doing so, they pointed to examples where women who had worked very hard to achieve target glucose levels prior to the trial had found it particularly psychologically challenging to delegate tasks such as the administration of basal insulin to the HCL:

“Some women … they're so used to doing everything themselves … (that) they aren't able to give up that part.” (HP‐018)

They further reported how such women had sometimes intervened in ways that may have impeded the system's functioning or algorithmic learning; for example, by constantly making adjustments, or overriding the system, through excessive use of corrective doses or the ‘Boost’ function (see Table 2). Interviewees also reported having had to work hard to address these women's anxieties and concerns; and, how their efforts to counsel and reassure them had sometimes been unsuccessful. Specifically, some interviewees pointed to examples where a few women had discontinued HCL early on in the trial, due to their difficulties delegating glycaemic management tasks to the system:

“we did have one participant who withdrew … because she couldn't tinker with it essentially. Because she wanted to be able to influence it … She just felt uncomfortable … She liked to be able to give extra boluses, and adjust more things than she could.” (HP‐003)

Such women, as interviewees further noted, had been used “to having tight glucose levels” (HP‐017) and had felt that the algorithm had not been “as aggressive as [they] would have liked” (HP‐07).

### 3.5 Candidacy and (difficulties) predicting who gains greatest benefit

Interviewees expressed the view that all women who used the HCL during the trial (excepting those who withdrew) experienced some clinical (e.g., increased time‐in‐range) and/or quality‐of‐life benefits (e.g., less work, fewer worries, better sleep):

“the overwhelming theme is that everyone does better with it, than without. Like definitely, a hundred per cent.” (HP‐005)

This, they suggested, applied even to those women who they perceived to have struggled to consistently implement management practices supportive of optimal closed‐loop performance:

“one of our patients … it all looked pretty haphazard, (and) we had to keep reminding ourselves that before she went on to closed‐loop she was 40% in target. And when she went on the closed‐loop, she was 60% in target. So … significant improvement.” (HP‐009)

Indeed, interviewees surmised that some such women had benefitted not only because the HCL had relieved them of responsibility for basal insulin administration, but also because it had reduced the detrimental impacts of, for example, not administering bolus doses correctly:

“We had one of our very early participants and I think she probably didn't use it that well, and I think possibly had she not had the closed‐loop, she would have [had] a very different outcome. Because it did pick up all the rubbish of her not correcting properly or not injecting, not carb counting, erratic eating patterns. And I think the closed‐loop really softened that blow.” (HP‐019)

Interviewees stressed, however, that while the HCL could compensate for small lapses or errors (e.g., by ramping up basal insulin delivery in response to rising glucose levels), it was unable to offset fully missed, mis‐timed, or miscalculated boluses (see Table 2).

#### 3.5.1 Difficulties predicting who would benefit

Due to pressures to meet trial recruitment targets, most interviewees described taking an inclusive approach to recruitment, including inviting women who they felt might struggle to use diabetes technology:

“we didn't want to prejudge who would be suitable for it… we thought, ‘we're gonna ask everybody who meet these [inclusion] criteria’ rather than thinking ‘oh well they're not good with technology, we better not’.” (HP‐016)

Reflecting on their experiences of supporting such women during the trial, they pointed to examples where their preconceived ideas about how effectively specific women would work with the HCL had been challenged. Some described being reasonably confident that they could predict who would “over‐engage” (HP‐015) with the HCL (typically, women who had micro‐managed their diabetes before the trial). However, most described finding it harder to second‐guess which women would under‐engage, with some noting that pregnancy itself could change behaviour radically:

“there was one woman in particular who was pretty hopeless at looking after diabetes outside of pregnancy and has done very, very well… We thought, ‘Should we be putting her in the trial?’ We did, because she met the criteria. And we were slightly nervous, thinking, ‘Well, this could be a disaster, maybe she'll just get frustrated and pull out of the trial’… But no, she didn't.” (HP‐009)

Interviewees surmised that women, such as the one described above, had engaged with the technology, and benefited from it, as it automated some of the tasks women had previously struggled to undertake (such as checking and correcting high glucose). Indeed, a few suggested that HCL provisioning could make constituent technologies, in particular insulin pumps, more accessible to a wider group of women:

“it's certainly easier having a woman on closed‐loop, than it is having a woman on a pump separately, because … you have to know a lot more about pumps … to make them work, than you do about a pump used in a closed‐loop system. So, if people are worried about insulin pumps, then actually closed‐loop is easier, and safer, than a pump used in a stand‐alone system.” (HP‐003)

#### 3.5.2 Use of HCL systems in routine clinical care

Given their perception that virtually all women benefited from using the HCL, and the difficulties predicting how individuals would engage with the technology, most interviewees suggested that all pregnant women be given opportunities to use a HCL in routine clinical care (see Table 2). In doing so, interviewees emphasised that women with well‐managed diabetes in early pregnancy (such as those who did not meet trial inclusion criteria) should also be included in clinical guidelines, because, while they might only gain modest clinical benefits, the technology could offer significant quality‐of‐life benefits:

“Some people have very good control because they're doing an amazing job of managing their sugars, and actually their life could be easier if they had this system … you know, some people do get up at ridiculous o'clock to give themselves extra insulin, et cetera, et cetera, and maintain great control that way. And a system that would support that, without their… intervention, would make their life much easier.” (HP‐015)

However, a minority did express concerns that HCL use in some women with well managed‐diabetes who “like to tinker with things” (HP‐017) might cause some frustration and/or anxiety (see Table 2).

In offering endorsement for an inclusive approach to HCL rollout in pregnancy, some also suggested that where the NHS was already funding CGM and pump technology, the additional cost of providing access to a HCL would be minimal, and, indeed, it would be “almost criminal” (HP‐014) not to meet this.

## 4 DISCUSSION

Healthcare professionals highlighted multiple clinical and quality‐of‐life benefits to using an HCL in pregnancy. While they attributed some of these benefits to the CGM component, they emphasised that the HCL conferred additional benefits. Healthcare professionals noted that, to secure maximum gains, an effective, three‐way collaboration between themselves, the woman and the HCL was required. As well as emphasising their own role in this collaboration, healthcare professionals noted that effective collaboration required women to interact with the HCL sufficiently, but not excessively; an issue that, as they further observed, some women had found challenging. Even when healthcare professionals felt that women had not achieved this balance, they still highlighted some benefits to HCL use. Some described having preconceived ideas about how well specific women would engage with the HCL, which were challenged during the trial. In light of their trial experiences, healthcare professionals favoured an inclusive approach to HCL rollout in routine clinical care. It is encouraging, therefore, that since these healthcare professionals were interviewed the National Institute for Health and Care Excellence (NICE) has recommended HCL as an option for managing blood glucose levels in type 1 diabetes in pregnant women.^27^

According to these healthcare professionals, benefits specific to using CGM included: provisioning of better information to inform diabetes management decisions; and, alarms facilitating use of tight(er) pregnancy glucose targets (for example, by mitigating women's worries about hypoglycaemia). Research involving other groups of CGM users^28^, ^29^ has identified similar benefits. Nevertheless, our own findings are important, since, to the best of our knowledge, no other qualitative studies have specifically explored CGM use in pregnancy from either healthcare professional or user perspectives. Moreover, our findings provide support for recent UK guideline recommendations^6^, ^7^, ^8^ that CGM be made available to all pregnant women with type 1 diabetes.

Healthcare professionals viewed the HCL as conferring additional benefits by relieving women of (some of) the burdens of diabetes management. Their perspectives reflect and reinforce findings from early phase trials, which have highlighted clinical and quality‐of‐life benefits.^13^, ^14^, ^15^, ^16^ Our findings also align with those of studies exploring the HCL experiences of other user groups.^9^, ^10^, ^11^, ^12^ Notably, however, while healthcare professionals noted the relative ease of use of this technology (as compared to stand‐alone pumps), they emphasised that, to attain maximum benefit, women still needed to undertake work, and collaborate actively with themselves and the HCL. In particular, they noted how women needed to know when to delegate management to the HCL and when to step in, e.g., by using the ‘Boost’ function and/or corrective doses. They also noted how some women had struggled to maintain this division of labour and had interacted little or too much with the HCL.

With regard to women who they perceived to have engaged with the HCL to a lesser extent than they would have recommended, healthcare professionals observed that, while not realising all the benefits the technology offered, these women still experienced *some* gains. Mirroring findings from research involving other user groups, such benefits arose partly from the system's ability to help mitigate the effects of missed, mis‐timed or miscalculated pre‐meal boluses.^30^ Moreover, healthcare professionals reported difficulties predicting how effectively women who had previously not engaged with diabetes self‐management would work with the HCL. This observation echoes findings from earlier research, which similarly highlighted healthcare professionals' difficulties determining who would gain (most) clinical benefit from using insulin pumps^18^ and HCL.^19^

With regard to women who were described as experiencing difficulties entrusting glycaemic management to the HCL, Kimbell et al. likewise observed how some caregivers of very young children struggled to transition to more passive management roles following HCL initiation.^31^ Notably, healthcare professionals in the current study observed that overly engaged women had often achieved near‐optimal glucose levels prior to the trial: there is a clear tension between this finding and the suggestion most made that *all* women be given opportunities to use a HCL in routine clinical care. Important questions remain as to: whether and how such individuals might be identified in advance; if they should then be counselled accordingly, discouraged, or excluded from HCL use; and/or if additional psychological support might be beneficial.

Finally, while healthcare professionals advocated for an inclusive approach to HCL access going forward, they emphasised that women would need a comprehensive package of professional support. Successful rollout/wider use of HCL technology is therefore likely to be contingent on adequate training and support being provided to healthcare professionals. Healthcare professionals' views about the training and support needed to support a national rollout are reported separately.^32^

### 4.1 Strengths and limitations

This is the first study to report healthcare professionals' experiences of supporting HCL use in pregnancy. The interviews were conducted by a highly experienced qualitative researcher, who worked hard to develop trust and rapport with interviewees. This led to the generation of rich and novel insights, with clear relevance to clinical practice. It is important, however, to recognise that interviewees expressed their opinions and views in the distinctive context of a confidential research interview. Hence, some may have used language that differed from that employed in their everyday clinical work. Indeed, some of the quotes we report use language which – taken out of context – may appear to some readers to be judgemental and/or lacking in understanding of the multi‐faceted challenges some women experience when managing type 1 diabetes in pregnancy. It should be remembered that such quotes were drawn from much longer transcripts, and it has only been possible to include material of direct relevance to our reporting in this article. We appreciate there are limitations to presenting quotes out of (their full) context. It is important to emphasise that, elsewhere in their interviews, all healthcare professionals shared experiences and perspectives which evidenced a strong and nuanced appreciation of the challenges involved in type 1 diabetes management and how these could be amplified by pregnancy and complex life circumstances.

It is also relevant to note that, as interviewees were involved in a clinical trial, they may have been technology enthusiasts; this may have been reflected in their appetite for a national HCL rollout. Furthermore, healthcare professionals' accounts were informed by experiences of supporting women who had chosen to participate in a clinical trial. Encouragingly, however, the AiDAPT trial successfully recruited women from diverse socio‐economic groups, and this may have been reflected in the diverse experiences of HCL use reported in this article. As women with HbA1c levels under 48 mmol/mol were ineligible for the trial, interviewees were unable to report experiences of supporting HCL use in this particularly tightly managed group; this may be an important group to consider in future research.

## 5 CONCLUSION

Interviewees were keen for HCL technology to be offered to all pregnant women with type 1 diabetes. They emphasised that, to gain maximum benefit, an effective, three‐way collaboration between themselves, the woman and the HCL was needed. Presenting HCL to pregnant women and healthcare teams as one pillar of a three‐party collaboration, and articulating the associated division of labour, may help promote optimal use of the technology in routine clinical care.

## AUTHOR CONTRIBUTIONS

JL conceived and designed the interview study with input from HRM. DR collected the data, which was then analysed by JL, DR, and RIH. JL conceived the concept for this article. RIH wrote the first draft, which JL then revised substantially with further input from RIH and DR. All authors reviewed, edited, and approved the final version of the manuscript.

## FUNDING INFORMATION

This project (NIHR 16/35/01) is funded by the Efficacy and Mechanism Evaluation (EME) Programme, an MRC and NIHR partnership. The views expressed in this publication are those of the author(s) and not necessarily those of the MRC, NIHR or the Department of Health and Social Care. Additional support for the artificial pancreas work has been provided by the National Institute for Health Research Cambridge Biomedical Research Centre, JDRF, and Wellcome Strategic Award (100574/Z/12/Z). Dexcom supplied discounted continuous glucose monitoring devices. Dr Tara Lee is funded by a Diabetes Research & Wellness Foundation Sutherland‐Earl Clinical Fellowship (reference SECF/21). Prof Rebecca M Reynolds acknowledges the support of the British Heart Foundation (RE/18/5/34216).

## CONSENT TO PARTICIPATE AND FOR PUBLICATION

All research participants provided written informed consent including for anonymized information to be published in this article.

## CONFLICT OF INTEREST STATEMENT

SH reports: receiving speaker & advisory board fees from Dexcom, Medtronic, Sanofi and Ypsomed; and being Director of ASK Diabetes Ltd., which receives consulting/training fees from CamDiab Ltd. RH reports: receiving speaker honoraria from Eli Lilly, Dexcom and Novo Nordisk; receiving license and/or consultancy fees from B. Braun and Abbott Diabetes Care; patents related to closed‐loop; and being Director at CamDiab. HRM sits on the Medtronic European Scientific Advisory Board and reports receiving speaker honoraria from Dexcom, Abbott, Medtronic and Novo Nordisk. JL, DR, TL, ARD, RMR, and RIH have no conflicts of interest to declare.

## Supporting information


Appendix S1.

## Data Availability

The datasets generated and analysed in the course of this study are not publicly available due to risks to individual privacy. However, they are available, via the corresponding author, on reasonable request.
