# Supplementary material for: Healthcare professionals' views about how pregnant women can benefit from using a closed‐loop system: Qualitative study
Source: Diabet Med. 2023 Mar 7;40(5):e15072. doi: 10.1111/dme.15072 (PMC10947358; doi:10.1111/dme.15072)
Supplement: Supplementary file 1 — Appendix S1. [file DME-40-0-s001.docx]

**SUPPLEMENTARY APPENDIX**

**AiDAPT Collaborative Group membership**

Katharine Hunt, Helen Rogers, King’s College Hospital, London, UK.

Damian Morris, Duncan Fowler, Josephine Rosier, Zeenat Banu, Sarah Barker, Gerry Rayman, Ipswich Hospital NHS Trust, Ipswich, UK.

Eleanor Gurnell, Caroline Byrne, Andrea Lake, Katy Davenport, Jeannie Grisoni, Shannon Savine, Cambridge University Hospitals NHS Foundation Trust, Cambridge, UK.

Helen Murphy, Tara Lee, Tara Wallace, Alastair McKelvey, Elizabeth Turner, Nina Willer, Norfolk and Norwich University Hospital, Norwich, UK.

Corinne Collett, Mei-See Man, Emma Flanagan, Matt Hammond, Lee Shepstone, Norwich Clinical Trials Unit, Norwich, UK.

Anna Brackenridge, Sara White, Anna Reid, Olanike Okolo, Guys and St Thomas’ NHS Foundation Trust, London, UK.

Eleanor Scott, Del Endersby, Leeds Teaching Hospitals NHS Trust, Leeds, UK.

Anna Dover, Frances Dougherty, Susan Johnston, Rebecca Reynolds, Royal Infirmary of Edinburgh, Edinburgh, UK.

Robert Lindsay, David Carty, Sharon Mackin, Isobel Crawford, Ross Buchan, Glasgow Royal Infirmary, Glasgow, UK.

David McCance, Louisa Jones, Joanne Quinn, Belfast Health and Social Care Trust, Belfast, Northern Ireland. Sarah Cains, Goher Ayman, Patient and Public Involvement (PPI) leads.

Julia Lawton, David Rankin, Ruth Hart, Barbara Kimbell, Mia Nelson, University of Edinburgh, Edinburgh, UK.
